# MiBiOmics: an interactive web application for multi-omics data exploration and integration

**DOI:** 10.1186/s12859-020-03921-8

**Published:** 2021-01-06

**Authors:** Johanna Zoppi, Jean-François Guillaume, Michel Neunlist, Samuel Chaffron

**Affiliations:** 1 INSERM, TENS, Université de Nantes, Nantes, France; 2 CHU Nantes, Inserm, CNRS, SFR Santé, Inserm UMS016, CNRS UMS 3556, Université de Nantes, 44000 Nantes, France; 3 CNRS UMR6004, LS2N, Université de Nantes, 44000 Nantes, France; 4 Research Federation (FR2022) Tara Oceans GO-SEE, Paris, France

**Keywords:** Multi-omics, Ordination, Biological networks, Data integration, R shiny

## Abstract

**Background:**

Multi-omics experimental approaches are becoming common practice in biological and medical sciences underlining the need to design new integrative techniques and applications to enable the multi-scale characterization of biological systems. The integrative analysis of heterogeneous datasets generally allows to acquire additional insights and generate novel hypotheses about a given biological system. However, it can become challenging given the often-large size of omics datasets and the diversity of existing techniques. Moreover, visualization tools for interpretation are usually non-accessible to biologists without programming skills.

**Results:**

Here, we present MiBiOmics, a web-based and standalone application that facilitates multi-omics data visualization, exploration, integration, and analysis by providing easy access to dedicated and interactive protocols. It implements classical ordination techniques and the inference of omics-based (multilayer) networks to mine complex biological systems, and identify robust biomarkers linked to specific contextual parameters or biological states.

**Conclusions:**

MiBiOmics provides easy-access to exploratory ordination techniques and to a network-based approach for integrative multi-omics analyses through an intuitive and interactive interface. MiBiOmics is currently available as a Shiny app at https://shiny-bird.univ-nantes.fr/app/Mibiomics and as a standalone application at https://gitlab.univ-nantes.fr/combi-ls2n/mibiomics.

## Background

The multi-scale characterization of biological systems is extending our knowledge about the functioning of organisms and natural ecosystems. Today, their multi-omics characterization is becoming standard, thus novel methodologies and easily accessible tools are required to facilitate the study of associations and interactions within and across omics layers [e.g. (meta-)genome, (meta-) transcriptome, metabolome] and scales (e.g. cells, organs, holobionts, communities). The analysis of single omics datasets has helped to identify molecular signatures associated to phenotypes of interest [1]. However, it usually does not allow to predict mechanisms underlying phenotypic variabilities [2]. Although multi-omics information is not sufficient to identify causes and consequences of a biological process, it can contribute to delineate key players sustaining it [3]. Indeed, exploring a biological system across several omics layers enable to capture additional sources of variability associated with a variation of interest and potentially to infer the sequence of events leading to a specific process or state [4]. Within the last decade, multi-omics integrative approaches have been applied across various fields including microbial ecology [5], genetics [6] and personalized medicine [7]. As of today, several integrative methods have been developed, but are often specific to a given experimental design, data type or a precise biological question [8]. Indeed, tools such as MONGKIE [9], are based on prior knowledge and integrate data by projecting them on known metabolic networks and biological pathways. More generally, existing multi-omics pipelines are focusing on certain data types (Metabolomics with MetaboAnalyst [10]) or on disease-related mechanisms (MergeOmics [11]). More widely applicable methods exist, such as the R package mixOmics [12] that provides several semi-supervised methodologies often based on ordination techniques. Considering the multiplicity of existing techniques, the selection of an appropriate workflow is challenging for biologists, especially when it comes to the representation of several system-level omics layers and its interpretation. There is a clear need for accessible (web) tools to facilitate the integration, analysis and representation of multi-omics datasets through an intuitive and guided approach.

MiBiOmics aims to provide established and novel techniques to reveal robust signatures in high dimensional datasets [13] through a graphical user interface allowing to perform widely applicable multi-omics analyses for the detection and description of associations across omics layers. Available as a web-based and a stand-alone application, it gives access to several R packages and tools to help users who are not familiar with programming to load and explore their data in a simple and intuitive way. MiBiOmics allows the parallel study of up to three omics datasets, as well as the in-depth exploration of each single dataset. It also provides easy access to exploratory ordination techniques and to the inference of (multilayer) correlation networks enabling useful dimensionality reduction and association to contextual parameters. The user can then compare results from these different approaches and cross-validate multi-omics signatures to generate confident novel hypotheses.

## Implementation

**Fig. 1:**
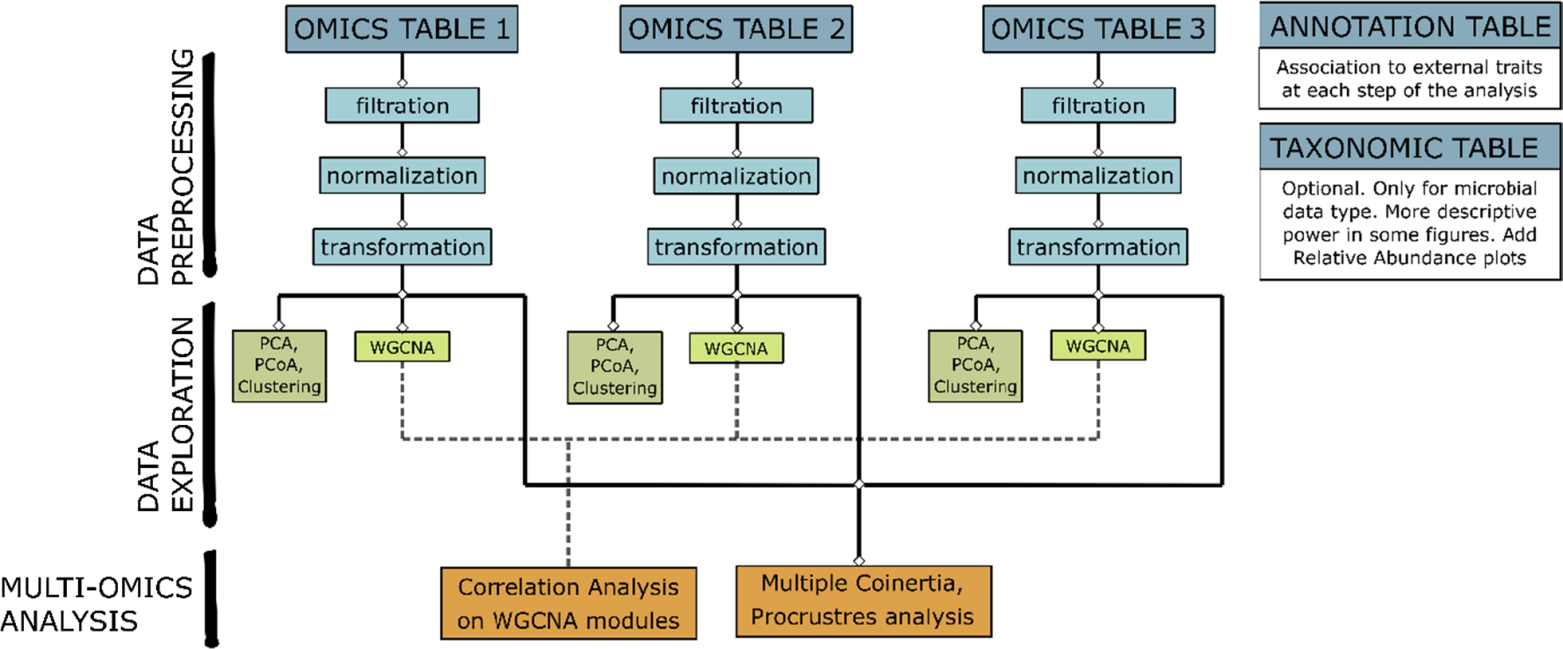
The MiBiOmics framework. The MiBiOmics workflow can be divided into three main tasks: data preprocessing, data exploration, and multi-omics integration. The data preprocessing task is dedicated to data upload, data filtration, normalization, and transformation. The data exploration task implements classical clustering methods, PCA, PCoA and WGCNA correlation networks that can be applied to each omics dataset separately. Finally, the multi-omics integration task allows the user to perform multi-omics exploration, integration and analyses using ordination techniques (multiple co-inertia and Procrustes analysis), and multi-omics network inference

MiBiOmics is implemented in R (Version 3.6.0) as a Shiny app providing an interactive interface to perform each step of a single- or multi-omics data analysis (Fig. 1). MiBiOmics is also accessible as a standalone application that can be easily installed via Conda (Version 4.6.12). The application is divided into five sections as described below:

### Data upload

Within MiBiOmics, the user can upload up to three omics datasets, allowing the data exploration and network analysis of a single- or multi-omics dataset. There must be common samples between omics datasets in order to perform all analyses provided by the application. An annotation table describing external parameters (e.g. pH, site of extraction, physiological measures) needs to be provided. These parameters may be quantitative or qualitative, and available for each sample. An additional taxonomic annotations table can be uploaded when one omics table corresponds to microbial lineages [e.g. as Operational Taxonomic Units (OTUs) or Amplicon Sampling Variants (ASVs)].

Following data upload, the user can filter, normalize and transform each data matrix using common methods, such as the center log ratio (CLR) transformation to deal with the compositional nature of sequencing data, or filtration based on prevalence. In this section, it is also possible to detect and remove potential outlier samples.

To allow new users to easily test the functionality of MiBiOmics, we provide two example datasets: the breast TCGA datasets from *The Cancer Genome Atlas* [14] allows to explore associations between miRNAs, mRNAs and proteins in different breast cancer subtypes; and a dataset from the *Tara* Oceans Expeditions [15, 16] to explore prokaryotic community compositions across depth and geographic locations.

### Data exploration

In this section, two ordination plots [Principal Component Analysis (PCA), Principal Coordinates Analysis (PCoA)] [17] are dynamically produced to visualize and explore relationships between samples, and to identify main axes of variation in each dataset. When OTUs or ASVs are uploaded with their taxonomic annotations, it is possible to obtain a relative abundance plot describing the proportion of lineages at a given taxonomic level (e.g. Phylum, Family, Genus or Species) in each sample.

### Network inference

The network inference section allows to perform a Weighted Gene Correlation Network Analysis (WGCNA [18]). Help sections are available to assist the user with parametrization, notably for optimizing the scale-free topology of the network. Here, WGCNA networks can be inferred for each uploaded omics dataset. We strongly advise users to read the WGCNA original publication and associated tutorials for this step of the analysis.

### Network exploration

**Fig. 2:**
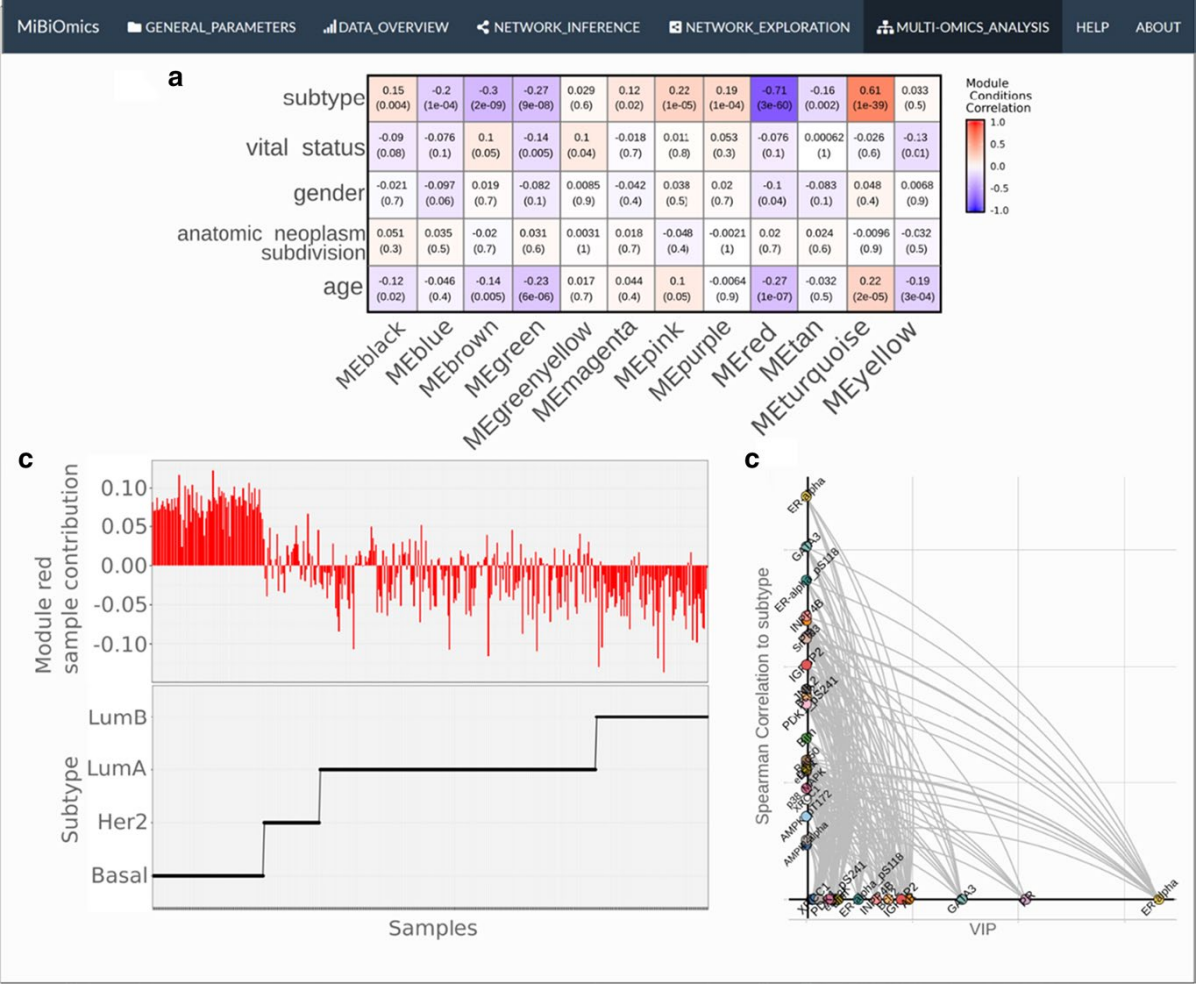
MiBiOmics networks exploration. MiBiOmics networks analysis visualizations (network exploration section) from the analysis of the The Cancer Genome Atlas Network [14] breast cancer TCGA datasets. **a** Correlation heatmap displaying associations of interest between mRNA’s WGCNA modules and contextual parameters. **b** The upper panel indicates the contribution of each sample in the red mRNA module delineation. Module eigenvalues are calculated for each sample and indicate how much they participate in the inference of each module. The lower panel indicates the corresponding subtype value for each sample. Here, Basal samples positively contribute to module red of mRNA, while Her2, LumA and LumB negatively contribute to the mRNA red module. **c** Hive plot displaying the protein red module's features according to their VIP scores, correlations to the subtype parameter and their relationships. In this hive plot, each point represents a variable of the protein WGCNA red module ordered, on the x-axis according to its VIP score, and on the y-axis according to its correlation to the subtype parameter. Edges linking proteins represent the actual edges of the WGCNA network. This representation is useful to distinguish central variables in the module (associated to many other variables) and predictive variables (features with a high VIP), and thus to assert whether central variables of the module are more associated to changes in the discriminant trait compared to predictive variables

The network exploration section allows to compute and explore significant associations between subnetworks or modules (e.g. of genes, transcripts, metabolites), and communities (of lineages) delineated from each omics layer, which contain highly correlated features. Each module is associated to all external parameters provided in the annotation table and correlations are visualized as a heatmap (Fig. 2a). Modules associated to parameters of interest can be further analyzed. The user can also identify which samples are contributing the most to the delineation of a specific module (Fig. 2b), a method provided by the WGCNA R package, which computes modules eigenvalues and allows to quantify the relative contribution of a given sample to the inference of a module. In case an OTUs/ASVs table is provided with taxonomic annotations, the relative abundance of lineages contributing to each module can be visualized as bar plots.

In addition, OPLS (Orthogonal Partial Least Square) regressions [19] can be performed using a selected module component as features in order to estimate its capacity to predict a given contextual parameter, and are useful to cross-validate a module-parameter association. The results of this analysis are represented as hive plots with two axes. On the x-axis, the module features are ordered according to their Variable Importance Projection (VIP) score (a measure of their weight in the OPLS regression), while on the y-axis they are ordered according to their correlations to an external parameter of interest (Fig. 2c).

### Multi-omics analysis

**Fig. 3:**
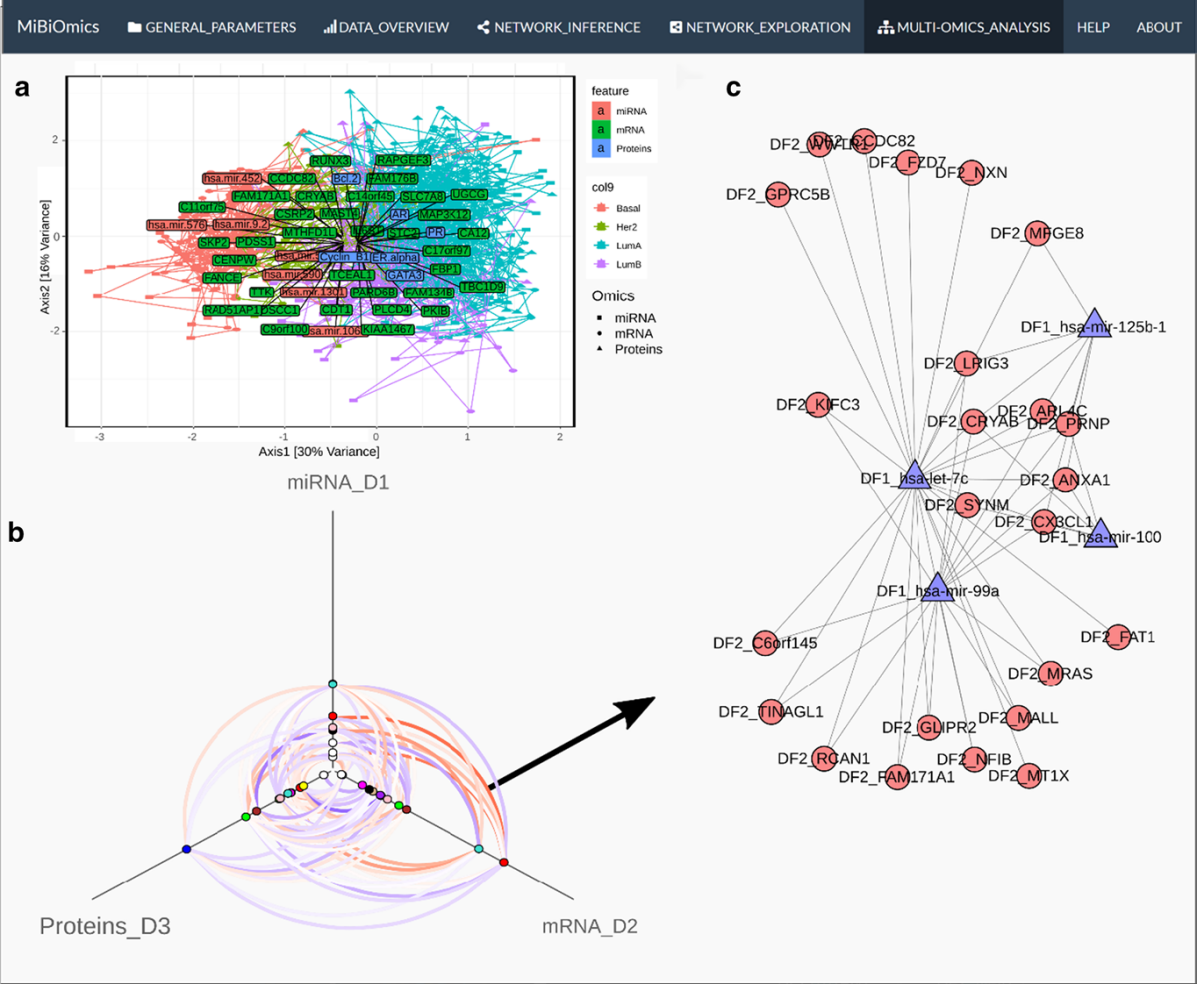
MiBiOmics multi-omics integration. MiBiOmics visualizations (multi-omics integration section) from the analysis of the The Cancer Genome Atlas Network [14] breast cancer TCGA datasets. **a** A multiple co-inertia plot integrating 3 omics layers and extracted miRNA, mRNA and protein drivers. In the MiBiOmics 3-layers co-inertia analysis representation, each sample is represented by a triangle: the three vertices are the positions of the sample in each ordination space of the 3-layers co-inertia analysis. The edges linking these vertices indicate how each layer of the sample’s covariate in each ordination space [27]. **b** Hive plot displaying modules of each omics network and their associations. Red edges represent positive associations, and blue edges negative associations. Edge color intensity reflects the correlation strength. **c** Bipartite network between mRNA features of the red mRNA module and miRNA features of the red miRNA module (Spearman Correlation > 0.35). When the user selects two modules of interest, variables belonging to both modules are correlated 2-by-2 and variables correlating significantly above a correlation threshold selected by the user are linked together with an edge

Here, MiBiOmics allows users to detect and study associations across omics datasets. Multivariate statistical tools including Procrustes analysis [17] and multiple co-inertia [20] are useful to compute and visualize the main axes of covariance, to extract multi-omics features driving this covariance, and to assert how the distribution of multi-omics sets can be compared. This central section of MiBiOmics implements an innovative approach for detecting robust links between omics layers. Building upon the WGCNA pipeline we innovate here by providing an applied methodology to link groups of variables from different omics nature to external variables capturing a trait of interest. To do so, all modules delineated within each omics-specific network are associated to each other by directly correlating their eigenvectors. Here, the dimensionality reduction of each omics dataset through module definition ensures a small number of correlations, thereby increasing the statistical power for detecting significant associations between omics layers. For visualization, a hive plot helps summarizing significant associations between each module as a multilayer network integrating links between omics-specific modules as well as their association to contextual parameters (traits or phenotypic characteristics). In this hive plot, each axis represents the network of a given omics layer. Corresponding modules are ordered on the axes according to their association to a contextual parameter of interest selected by the user. Modules with no significant associations are not depicted. Significant associations between omics-specific modules are represented, and individual associations between modules can also be visualized as heatmaps and data frame. Conveniently, the user can also select modules of interest to investigate pairwise correlations between modules' features and delineate groups of modules associated together and to an external parameter of choice. Following the identification of multi-omics modules related to a parameter of interest, the user can further investigate the pairwise correlations between variables of both modules inferred from different omics layers through the bipartite network represented in Fig. 3c or with the correlation heatmap.

Herein, we developed and implemented a novel multi-omics integration tool called multi-WGCNA. By reducing the dimensionality of each omics dataset in order to increase statistical power, multi-WGCNA is able to efficiently detect robust associations across omics layers. In addition, these multi-omics associations are linked to external traits (categorical or continuous) into a network of features for extracting robust biomarkers. We also implemented new visualization graphics to represent these multi-omics associations, an important addition in our opinion since representing multilayer associations is often challenging. Importantly, all figures generated by the application (PCA, PCoA, relative abundance plots, WGCNA outputs, hive plots, multiple co-inertia, Procrustes plots, correlograms, bipartite networks) can be downloaded (as svg or pdf files), as well as network features as csv files (WGCNA modules information, eigenvalues and co-inertia drivers).

## Results and Discussion

MiBiOmics enables the exploration, integration, analysis and visualization of up to three omics datasets. Through the primary exploration of a dataset, the inference of biological networks and the extraction of multi-omics associated features, the application provides a ready-to-use analysis pipeline to interactively explore sources of variability and variables of interest in a given biological dataset, as well as associations between multi-omics features in multi-scale studies.

The inference of networks from omics features is useful to represent and model the complex architecture of putative interactions in biological systems. In addition, networks provide a way to reduce the dimensionality of a dataset by delineating cohesive groups of co-varying, often functionally related features, that can then be associated to contextual or phenotypic characteristics of interest [3]. A key functionality of MiBiOmics is the multi-omics adaptation of WGCNA [18] to explore association across omics datasets via a network-based approach. As shown in Fig. 2a, the interface provides the ability to interactively probe associations in each omics layers of different breast cancer subtypes [14] within each network and their association to patient parameters. We further used these associations to external parameters to infer relation across multi-omics modules. The original WGCNA outputs are provided by the application to deepen the analysis between modules and external parameters (Fig. 2b). In addition, we provide the user with the possibility to perform an OPLS regression for modules of interest to evaluate the robustness of these variables to predict a given trait or phenotype. Figure 2c is an example of an OPLS regression using WGCNA module variables as features. On the x-axis the features of the red module are ordered according to their VIP score (their importance for the module), and on the y-axis according to their correlation to the subtype parameter. This figure highlights how central features of a WGCNA module relate to an external parameter.

The exploratory multi-omics analysis allows to study the main axes of covariance across omics profiles and give the ability to discover and select variables implicated in an association between omics datasets. The concomitant application of (multiple) co-inertia (Fig. 3a) and/or Procrustes multivariate techniques with the exploration of multi-omics correlations between WGCNA modules of distinct omics layer (Fig. 3b), provides a complementary vision of multi-omics relationships. The MiBiOmics interface allows to explore WGCNA modules of interest to directly infer significant associations between features from distinct omics layers (Fig. 3c). In a multi-omics adaptation, WGCNA can be used to delineate a group of modules associated together and to a parameter of interest and extract features of different omics nature but related to each other. While an interactive version of WGCNA already exists [21], MiBiOmics goes beyond by providing a multi-omics strategy to identify correlated modules across omics layers and generate novel hypotheses. Associating modules across different datasets has already been performed in the original WGCNA article [18] and reproduced in several studies. For example, the overlap of modules between transcriptional profiles of different tissue [22] was assessed, as well as a comparison between proteomics and gene expression profile of modules in a cohort of Alzheimer patients [23]. In both cases, the association between modules was determined by overlapping identical features (e.g. same genes in a given reference genome) within each module, a method which is not applicable when omics datasets do not contain similar data types or refer to the same biological system. In MiBiOmics, we enable the inference of relationships between omics layers within an entire biological system (e.g. holobiont) or ecosystem (e.g. the plankton), which makes it more widely applicable and especially suited for omics-based environmental studies.

**Fig. 4:**
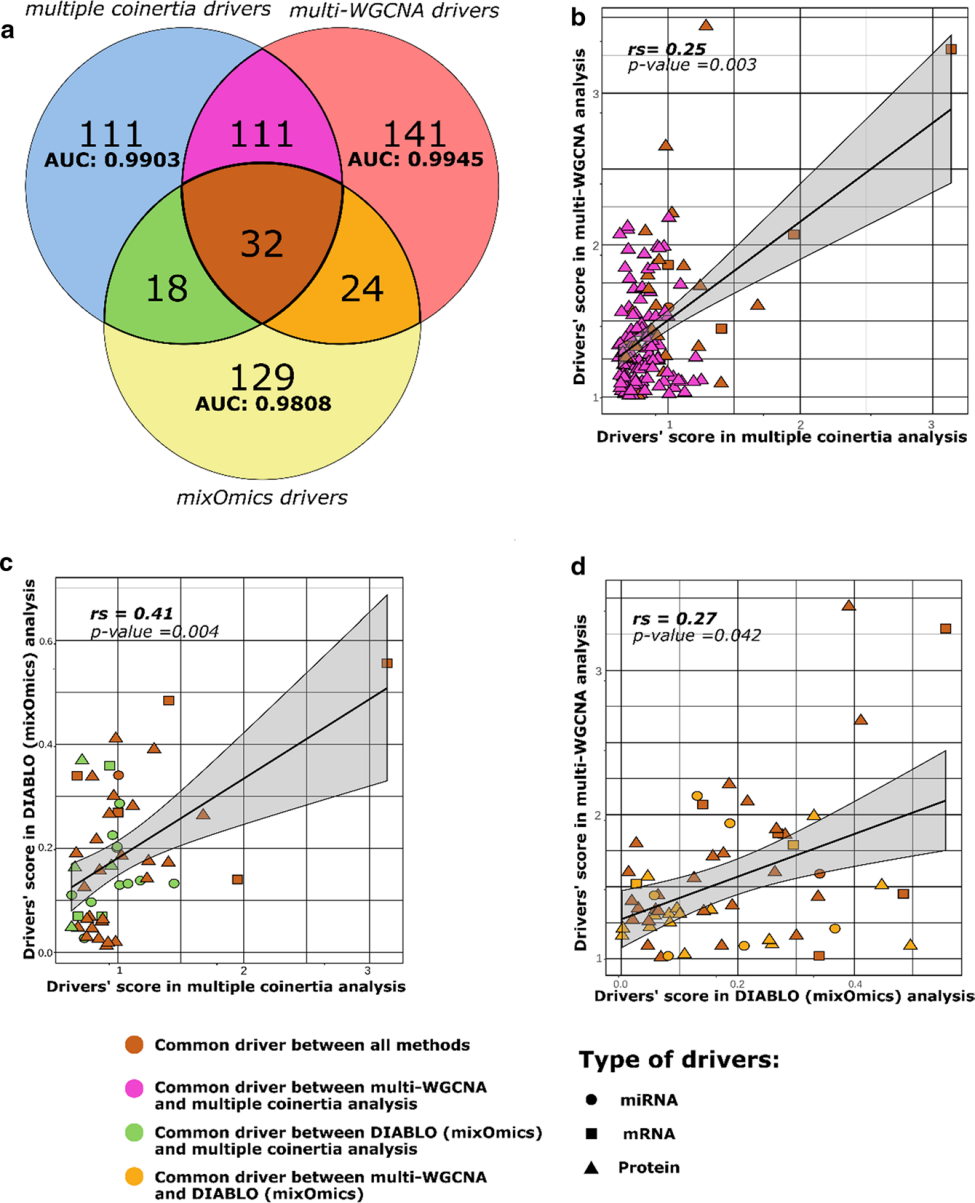
MiBiOmics and mixOmics comparison. **a** Venn diagram displaying common and distinct features extracted by DIABLO (mixOmics), multiple co-inertia and multi-omics WGCNA (MiBiOmics). Area under the curve (AUC) scores were computed to compare features selection and model performance of each method. **b** Selected features' weights comparison between multiple co-inertia and multi-WGCNA on their common subset of features. **c** Selected features' weights comparison between multiple co-inertia and DIABLO (mixOmics) on their common subset of features. **d** Selected features' weights comparison between DIABLO (mixOmics) and multi-WGCNA method on their common subset of features

We compared methods integrated in MiBiOmics (see Additional file 1 for details) to the mixOmics DIABLO methodology [24]. Within MiBiOmics, the multiple co-inertia analysis and the multi-WGCNA procedures provide the user with two integrative and exploratory methods, which can be applied to any type of data, and associated to not only categorical traits, but also quantitative traits. To highlight the complementarity of our application with DIABLO, we performed an in-depth comparison of biomarkers extracted by each method when analyzing the TCGA dataset. Only few multi-omics features associated to breast cancer subtypes in the TCGA dataset were extracted by all three methods (n = 32, Fig. 4a). Both methods integrated in MiBiOmics (i.e. multiple co-inertia and multi-omics WGCNA) and DIABLO extracted mostly distinct features (Fig. 4a) underlining the probable complementarity of these multi-omics integrative strategies. Scores attributed by each method to the common set of extracted features were also dissimilar (Fig. 4b–d and Additional file 1: Table S1). This may be explained by the fact that these methods implement fundamentally different approaches to features extraction and selection, which confirms the complementary nature of each analysis. For comparing the predictive power of models integrating features extracted by each method, we performed Sparse Partial Least Square Discriminant Analysis (sPLS-DA) and computed the corresponding mean AUC scores (Fig. 4a and Additional file 1: Figure S1). All models can be considered to be highly predictive of the cancer subtype phenotypes, with the miBiOmics multi-omics WGCNA methodology obtaining the highest AUC score (AUC = 0.9945), while the multiple co-inertia analysis performed very well too (AUC = 0.9903). Features extracted by the DIABLO method from mixOmics resulted into a lowest score (AUC = 0.9808) but remained highly predictive. Generally, these methods may benefit from an enrichment method applied to the list of extracted drivers [20].

**Fig. 5:**
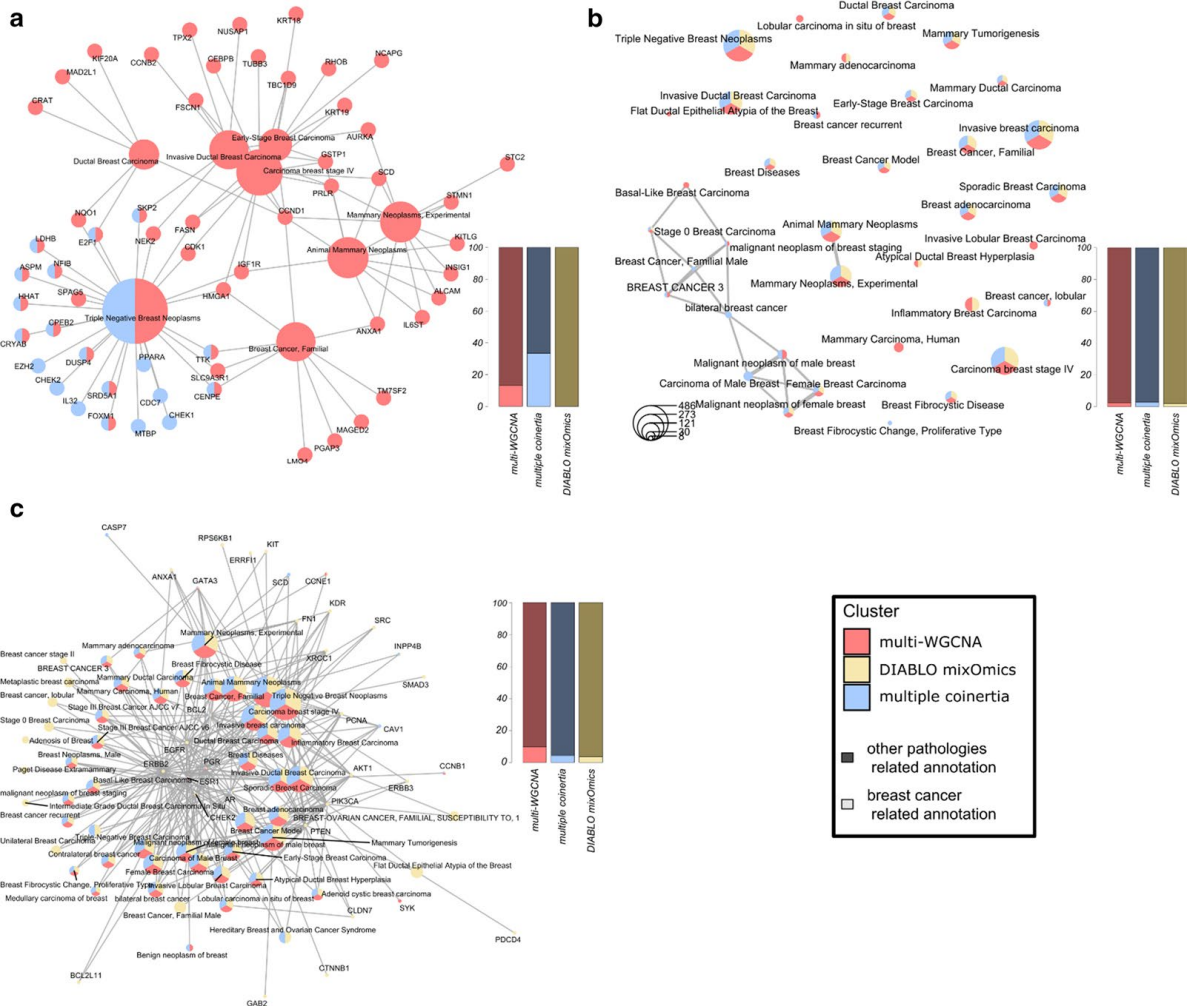
Comparison of extracted features by DIABLO (mixOmics), multi-WGCNA (MiBiOmics) and multiple co-inertia analyses (MiBiOmics). To compare the performance of each method, a gene enrichment analysis was performed using ClusterProfiler [28] and the DisGenNet (DGM) database [29]. **a** Diseases annotations from the DGM database and their corresponding genes associated to each subset of mRNA extracted features (DIABLO in yellow, multi-WGCNA in pink and Multiple co-inertia analysis in blue). **b** Diseases annotations from the DGM database associated to each subset of validated targeted genes by miRNA extracted features (DIABLO in yellow, multi-WGCNA in pink and multiple co-inertia analysis in blue). **c** Disease annotations from the DGM database and their corresponding proteins associated to each subset of protein extracted features (DIABLO in yellow, multi-WGCNA in pink and Multiple co-inertia analysis in blue). In each plot, the side bar plot indicates the proportion of breast cancer related annotations compare to the other pathologies associated terms

Through a gene-disease functional enrichment analysis (see Additional file 1), only the multi-WGCNA and multiple co-inertia methods were able to extract several biomarkers significantly associated to breast cancer while DIABLO found no mRNA related to breast cancer (Fig. 5a). In proportion, MiBiOmics tools extracted more mRNAs related to several stage of breast cancer development or tumor type (Fig. 5a). Some of these terms, such as the Carcinoma breast stage IV, were only retrieved by mRNAs extracted via multi-WGCNA. Also, the results obtained with the multiple co-inertia were more specific with close to 40% of mRNAs related to breast cancer (Fig. 5a). We performed a similar analysis on extracted miRNA features by retrieving their targeted genes. Similarly, for subsets of validated gene targets, we performed a functional enrichment analysis to find their association to diseases (Fig. 5b). Here, most breast cancer associated terms were found by all three methods. Notably, both multi-WGCNA and multiple co-inertia analyses were also able to highlight specific annotations related to male disposition in breast cancer or basal-like phenotype of breast tumor. The ratio of breast cancer related terms against other pathologies related terms was low for all methods but may be explained by the generally wide targeting nature of miRNAs. The functional enrichment analysis on extracted proteins by DIABLO (mixOmics), multi-WGCNA and multiple co-inertia analysis (MiBiOmics) (Fig. 5c) was also performed, and most of the breast related annotations were found by all three methods. However, DIABLO extracted several proteins associated to additional terms related to different stage of breast cancer evolution, while multi-WGCNA extracted the highest proportion of breast cancer related proteins compare to other pathologies.

This comparison of disease related annotations of extracted features showed the complementarity of the three methods. While the analyses extracted mostly different features related to tumor type, all of them were found highly predictive of the tumor subtype and were often associated to the same disease. Features extracted exclusively by one of the three method also participated in the enrichment of specific breast cancer stage annotations, and highlighted the potential of these methods in complementing each other in the analysis and characterization of multi-omics associations. We also evaluated the potential of each method to extract mRNAs, miRNAs, and proteins related to breast cancer annotations by computing the accuracy, recall, and F1-score for each method and each omics data type (See Additional file 1 and Additional file 1: Table S1). The performance of each method was found dependent of the nature of the data and both pipelines performed differently in terms of accuracy to extract features associated to breast cancer. Overall, the multi-WGCNA approach was found more accurate with regards to the mRNA features extraction associated to breast cancer, while DIABLO was found more accurate in extracting proteins associated to breast cancer (Additional file 1: Table S1).

Overall, MiBiOmics provides two complementary methods to extract associated variables between omics layers and in relationship with a trait of interest. Both multi-WGCNA and multiple co-inertia analyses highlighted specific protein biomarkers that were not identified by DIABLO. For example, both multi-WGCNA and multiple co-inertia analyses highlighted specific annotations related to male disposition in breast cancer or basal-like phenotype of breast tumor. A more specific example is the identification of the SYK protein only by the multi-WGCNA method. The SYK protein appears to have a dual role: depending on the alternative splicing of the mRNA it may act as a pro-oncogene or a tumor suppressor protein, and can interact differentially with its targeted genes [25]. The mechanisms surrounding this dual role of the SYK protein are still being largely studied [26]. Here, the multi-omics hive plots and bi-partite networks provided by MiBiOmics can be useful to generate new hypothesis on the associations and potential interactions between SYK and specific genes and miRNAs linked in the multi-omics network. While MiBiOmics may be useful to generate new hypotheses about molecular processes, it cannot infer causal mechanisms between omics features and phenotypes. This would require experimental validations, which can actually be guided by MiBiOmics results. To provide an exploratory and integrative framework for multi-omics studies, MiBiOmics distinguishes itself by providing a powerful dimensionality reduction and unsupervised method combining both ordination and graph-based techniques, which enables to study complex biological systems as a whole. Importantly, it also integrates contextual information by linking multi-omics signatures to qualitative and quantitative contextual parameters.

## Conclusion

MiBiOmics is an interactive web-based (and standalone) application to easily and dynamically explore associations across omics datasets. Through an innovative network-based integrative strategy, it can help biologists to identify putative mechanisms of interactions and generate novel hypotheses. The core of the application lies behind the reduction of dimensionality across omics datasets to efficiently link them at the molecular level, and to identify biomarkers associated with a given trait or phenotype. The MiBiOmics pipeline facilitates the exploration, integration, and analysis of multi-omics datasets to a broad audience by providing scientists a powerful way to predict and explore putative molecular mechanisms underlying complex phenotypes across a wide range of biological scales and systems.

### Availability and requirements

Project name: MiBiOmics

Project home page: https://gitlab.univ-nantes.fr/combi-ls2n/mibiomics

Operating system(s): Platform independent

Programming language: R

Other requirements: for the local installation Conda 4.6.12 or Docker

License: AGPL-3

Any restrictions to use by non-academics: No restrictions

## Supplementary Information

**Additional file 1.** Supplementary material (methods, table S1, and figure S1).

## Data Availability

The datasets provided as example within MiBiOmics application are available in the data repository, at https://gitlab.univ-nantes.fr/combi-ls2n/mibiomics.

## Abbreviations

ASV: Amplicon sequence variant
AUC: Area under the curve
DGN database: Disease gene network database
DIABLO: Data integration analysis for biomarker discovery using latent variable approaches for omics studies
OPLS: Orthogonal partial least square
OTU: Operational taxonomic unit
PCA: Principal component analysis
PCoA: Principal coordinates analysis
sPLS-DA: Sparse partial least square discriminant analysis
TCGA database: The cancer genome atlas database
VIP: Variable importance projection
WGCNA: Weighted gene correlation network analysis
