# Supplementary material for: MiBiOmics: an interactive web application for multi-omics data exploration and integration
Source: BMC Bioinformatics. 2021 Jan 6;22:6. doi: 10.1186/s12859-020-03921-8 (PMC7789220; doi:10.1186/s12859-020-03921-8)
Supplement: Supplementary file 1 — Additional file 1. Supplementary material (methods, table S1, and figure S1). [file 12859_2020_3921_MOESM1_ESM.docx]

**Supplementary material for:**

**MiBiOmics: An interactive web application for multi-omics data exploration and integration**

Johanna Zoppi, Jean-François Guillaume, Michel Neunlist and Samuel Chaffron.

Details about multi-omics data analysis methodologies we compared in the article (Figure 4) are described below. Both methods implemented in MiBiOmics (multi-WGCNA and multiple co-inertia) were compared to DIABLO [1] integrated in the mixOmics R package.

**mixOmics DIABLO**

We performed a DIABLO analysis on the whole breast TCGA dataset to extract multi-omics features associated to the tumor subtype parameter. Following the DIABLO tutorial [1], we choose a design where all the omics blocks (mRNA, miRNA and proteins) are connected with a link of 0.1. For selecting the final model we choose the centroid distance with 4 components, and identified an optimum number of extracted features per components using the function tune.block.plsda. A total of 203 non-redundant features were selected using this protocol, which we compared with both methods implemented in MiBiOmics.

**MiBiOmics multi-WGCNA**

For the integration of multi-omics datasets, MiBiOmics allows the inference of multilayer networks based on the WGCNA methodology developed by Langfelder and Horvath in 2008 [2]. This multilayer network is built by detecting significant associations between WGCNA subnetworks or modules delineated for each omics dataset. In addition, association to contextual information is also integrated by detecting modules of the multilayer network significantly associated to a given trait or phenotype. To extract multi-omics features associated to a contextual parameter of interest (here the tumor subtype in the TGCA dataset), the following protocol was implemented:

- WGCNA signed networks are inferred for all omics datasets (miRNA, mRNA and protein datasets). Here, we used a bi-weight mid-correlation (or bicor), and choose soft powers of 16, 8 and 10 with a minimum module size of 4, 6 and 4 for the miRNA, mRNA and protein datasets, respectively. For these parametrization steps (soft power and minimum module size), we strongly advise users to follow protocols and instructions described in the WGCNA article [2].

- Modules associated to our trait of interest (tumor subtype) were selected based on the Spearman correlation (and associated p-value) between the parameter and the modules eigenvalue (abs(cor.) > 0.5 and p-value < 0.001). Based on these criteria, three modules were selected: the mRNA red and turquoise modules, and the protein green module.

- Starting from this first set of modules we delineated a group of modules significantly associated together. The hive plot in the MiBiOmics ‘multi-omics analysis’ section allows to visualize how eigenvalues of each module correlate to each other across omics layers. This step allows to detect significant associations between modules and thus between omics layers. Here, we selected modules associated to the first set of modules directly associated to the trait of interest (the red and turquoise from the mRNA network, and the green from the protein dataset; Spearman abs(cor.) > 0.5 and p-value < 0.001. Using this procedure, we obtained a multilayer network or network of modules associated to a given trait. At this stage, the following additional modules were selected: blue and turquoise from the miRNA network, brown, red and turquoise from the mRNA network and blue and green from the protein network.

- For each module, the list of features, their VIP scores, correlations to the subtype parameter and associated p-value were downloaded via the ‘Network Exploration’ tab, after setting the appropriate number of components for each sPLS-DA (the optimum number of components is identified by the first minimum local on the Root Mean Square Error of Prediction (RMSEP) plot.

- Given some modules may contain many features, we selected these features weighted by their importance in the module (based on the VIP score), and their association to the parameter of interest (tumor subtype). Here, we selected only features that obtained a VIP score above 1 and an associated p-value below 0.05.

Using this protocol, 308 features were selected across the mRNA, miRNA and protein datasets to be significantly related together and/or associated to the tumor subtype.

**MiBiOmics multiple co-inertia**

The multiple co-inertia analysis is an ordination technique that can be applied to multi-omics datasets, where separate ordinations are performed on each omics layers and then factorized together. The resulting factorization defines new scores to infer the covariance within each sample between omics datasets, which are then used as coordinates in the co-inertia representation to project and scale the samples on the two main axes of covariance. Using the TGCA multi-omics dataset, we performed a multiple co-inertia as implemented in MiBiOmics with the ade4 R package, and extracted drivers on the first axis of co-variance (we selected the first axis of the multiple co-inertia along which samples were ordered according to their respective subtype). These drivers or features are ranked according to how much they participate to the co-variance on this axis. Here, we selected the top 30% features with the highest absolute score in the first axis of the total covariance.

Following this procedure, a total of 272 multi-omics features were extracted.

**Comparing the predictive power of each method**

In order to compare the capacity of these methods to extract features associated to a parameter of interest we performed a Sparse Partial Least Squares Discriminant Analysis (sPLS-DA) using each method features associated to the tumor subtypes using the mixOmics *plsda* function. The appropriate number of components was chosen using the recommended value of the *perf* mixOmics function and the more accurate distance metric (the selected number of components for the sPLS-DA was 6, 7 and 3 for the multiple co-inertia, multi-WGCNA and DIABLO features, respectively). The AUC was computed, and ROC curves were plotted for each sPLS-DA (Figure S1) to estimate and compare the predictive power of each method according to the tumor subtype parameter. The AUC indicated a strong predictive power for all three methodologies (DIABLO-AUC = 0.973, multi-WGCNA-AUC = 0.999, multiple co-inertia-AUC = 0.990) but using distinct extracted features.

**Comparing the extracted biological features and their relation to breast cancer subtype**

Because each set of extracted features was found highly predictive of the breast cancer subtypes but different from each other, we analyzed their relationships to breast cancer annotation terms. We used the DGN (Disease Gene Network) database assisted with the *ClusterProfiler* R package to perform a functional enrichment analysis. For the mRNA and protein extracted sets, we recovered the corresponding entrezID and performed a functional enrichment independently on the subsets of mRNAs and proteins extracted by each method (DIABLO mixOmics, multi-WGCNA MiBiOmics, and multiple co-inertia MiBiOmics). For the miRNA, we first recovered their targeted genes and ran the analysis on the entrezID of these targeted genes.

We described the accuracy of each method by calculating the number of features related to breast cancer terms compare to the total number of features related to other pathologies. We also calculated accuracy, recall and F1-score to evaluate the performance of each method:

|  | Condition Positive | Condition Negative |
| --- | --- | --- |
| Predicted condition positive | **True Positive:** mRNA/miRNA/Protein **contributes** to at least to one breast cancer associated term and **was extracted** by the method. | **False Positive:** mRNA/miRNA/Protein **does not contribute** to at least to one breast cancer associated term and **was extracted** by the method. |
| Predicted condition negative | **False Negative:** mRNA/miRNA/Protein **contributes** to at least to one breast cancer associated term and **was not extracted** by the method. | **True Negative:** mRNA/miRNA/Protein **does not contribute** to at least to one breast cancer associated term and **was not extracted** by the method. |

$$F1-score= \frac{\boldsymbol{True Positive}}{\boldsymbol{True Positive}+ \frac{1}{2}(\boldsymbol{False Positive}+\boldsymbol{False Negative})}$$

$$Accuracy= \frac{\sum\boldsymbol{True Positive}\boldsymbol{+}\sum\boldsymbol{True Negative}}{\sum Total Population}$$

$$Recall= \frac{\sum\boldsymbol{True Positive}}{\sum Condition Positive}$$

The resulting F1-score, accuracy and recall values are listed in Table 1.

**References**

1. Singh, A., Gautier, B., Shannon, C.P., Vacher, M., Rohart, F., Tebbutt, S.J., Lê Cao, K.A.: DIABLO: anintegrative approach for identifying key molecular drivers from multi-omics assays. Bioinformatics35(17),3055–3062 (2019)

2. Langfelder, P., Horvath, S.: WGCNA: An R package for weighted correlation network analysis. BMCBioinformatics (2008). doi:10.1186/1471-2105-9-559


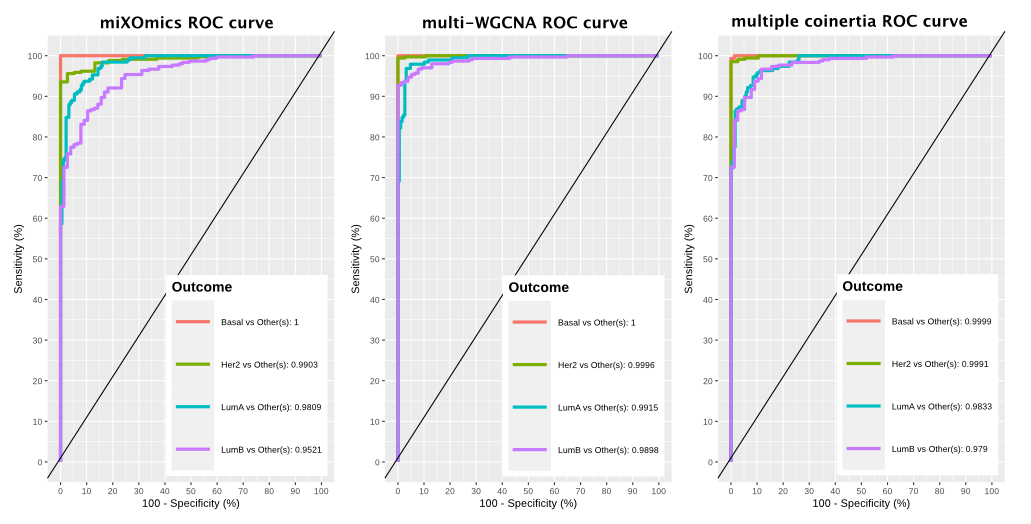


**Figure S1 : Predictive power of DIABLO (mixOmics), multi-WGCNA (MiBiOmics) and multiple co-inertia (MiBiOmics) for the tumor subtype parameter of the TCGA dataset.** ROC curves and AUC were obtained using sPLS-DA models built using extracted features associated to the tumor subtype parameter by the three methods (DIABLO from mixOmics, multi-WGCNA and multiple co-inertia from miBiOmics).

|  |  | DIABLO mixOmics | Multi-WGCNA | Multiple co-inertia |
| --- | --- | --- | --- | --- |
| F1 score | mRNA | 0 | 0.17 | 0.08 |
|  | miRNA (targeted genes) | 0.31 | 0.31 | 0.29 |
|  | Protein | 0.54 | 0.13 | 0.20 |
| Accuracy | mRNA | 0.77 | 0.72 | 0.73 |
|  | miRNA (targeted genes) | 0.20 | 0.27 | 0.33 |
|  | Protein | 0.55 | 0.39 | 0.41 |
| Recall | mRNA | 0 | 0.15 | 0.07 |
|  | miRNA (targeted genes) | 0.99 | 0.94 | 0.86 |
|  | Protein | 0.39 | 0.07 | 0.11 |

**Table S1 :** F1 score, accuracy and recall to evaluate the performance of each tool (DIABLO mixOmics, multi-WGCNA and multiple co-inertia MiBiOmics) in extracting features related to breast cancer annotations.
